# Grading of MRI–detected skull-base invasion in nasopharyngeal carcinoma with skull-base invasion after intensity-modulated radiotherapy

**DOI:** 10.1186/s13014-019-1214-3

**Published:** 2019-01-17

**Authors:** Yanru Feng, Caineng Cao, Qiaoying Hu, Xiaozhong Chen

**Affiliations:** Department of Radiation Oncology, Zhejiang Cancer Hospital, Hangzhou, 310022 China

**Keywords:** Nasopharyngeal carcinoma, Skull-base invasion, Intensity-modulated radiotherapy, Prognostic value, American joint committee on Cancer staging system

## Abstract

**Background:**

The aim of this study is to evaluate the prognostic value of grading MRI–detected skull-base invasion in nasopharyngeal carcinoma (NPC) with skull-base invasion after intensity-modulated radiotherapy (IMRT).

**Methods:**

This study is a retrospective chart review of 469 non-metastatic NPC patients with skull-base invasion. Patients were classified as extensive skull-base invasion (ESBI) group and limited skull-base invasion (LSBI) group.

**Results:**

Multivariate analysis showed that the skull-base invasion (LSBI vs. ESBI) was an independent prognostic predictor of progression free survival (PFS). The estimated 5-year local failure free survival (LFFS), distant metastasis free survival (DMFS), PFS, and overall survival (OS) rates for patients in the T3-LSBI and T3-ESBI group were 92.9% versus 93.5, 89.8% versus 86.1, 81.6% versus 76.4, and 93.5% versus 86.3%, respectively (*P* > 0.05).

**Conclusion:**

Grading of MRI-detected skull-base invasion is an independent prognostic factor of NPC with skull-base invasion. It is scientific and reasonable for skull-base invasion as a single entity to be classified as T3 classification.

## Introduction

Nasopharyngeal carcinoma (NPC) is endemic in China and over 33,000 new patients were diagnosed in 2012 [1]. According to the 8th edition of the American Joint Committee on Cancer (AJCC) staging system for NPC, T classification is based on the anatomical extent of the primary tumor and which has been proposed in the era of intensity-modulated radiotherapy (IMRT) [2, 3]. In the 8th edition of the AJCC staging system for NPC, skull-base invasion is classified as T3 disease [2].

With respect to the prognostic value of magnetic resonance imaging (MRI)-detected skull-base invasion for NPC, there are limited reports, especially, for patients treated by IMRT [4–6]. The aim of this study is to grade MRI–detected skull-base invasion in NPC with skull-base invasion and evaluate the prognostic value of the grading in the era of IMRT.

## Materials and methods

### Patients and patient workup

This study was approved by the Institutional Review Board to identify the patients diagnosed with NPC in our center. Because this study was a retrospective study, consent was not obtained and patient records were anonymized and de-identified prior to analysis. The medical records of consecutive 695 patients with previously untreated, biopsy-proven, non-metastatic NPC that was treated with IMRT between January 2007 and February 2012 in our center were retrospectively evaluated. Of these, 469 patients with skull-base invasions were included in this study. All patients were restaged according to the 8th edition of the AJCC staging system. The pretreatment workup included a complete history and physical examination, hematology, and biochemistry profiles, fiber-optic nasopharyngoscopy, MRI of the head and neck, bone scintigraphy, computed tomography (CT) scan of the chest and abdominal region, and dental check.

### MR imaging

All patients underwent MRI on a 1.5- or 3.0-T system (Magnetom Symphony/ Verio, Siemens Healthcare, Erlangen, Germany) with a head-and-neck combined coil. The scan range covered from the suprasellar cistern to the inferior margin of the sternoclavicular joint. All patients underwent T1 weighted and fat-suppressed T2 weighted sequences. After bolus injection of 0.2 ml/kg gadopentetate dimeglumine, contrast-enhanced T1-weighted images were obtained. Two radiologists independently evaluated all scans, and any disagreements were resolved by consensus.

Skull-base invasion was diagnosed using the following criteria: (1) a defect in the low signal intensity of the bone cortex on T1-weighted image and (2) high signal intensity marrow replacement by low signal intensity tissue on T1-weighted image (an obvious enhancement in the enhanced scan) [5]. Patients were classified as limited skull-base invasion (LSBI) group if they had invasion of one or more of these sites including the pterygoid process, base of sphenoid bone, petrous apex, clivus, and foramen lacerum.

Patients were classified as extensive skull-base invasion (ESBI) group if they had invasion of one or more of these sites including the medial pterygoid plate, foramen ovale, pterygopalatin fossa, foramen rotundum, foramen magnum, hypoglossal canal, lateral pterygoid plate and jugular foramen [4, 6].

### Treatment

All patients received definitive IMRT. A detailed description of IMRT has been previously reported [7]. Briefly, using the simultaneous integrated boost technique, the dose prescribed was 69–70.4 Gy, 63–67.2 Gy, 60–60.8 Gy and 54–54.4 Gy in 30–32 fractions delivered over 6 weeks at the periphery of the planning target volume (PTV) of primary tumor, PTV of metastatic lymph nodes, PTV of high-risk clinical target volume, and PTV of low-risk clinical target volume, respectively. Most patients (*n* = 459, 97.9%) received platinum-based neoadjuvant, concurrent, or adjuvant chemotherapy.

### Follow-up and statistical analysis

Follow-up was calculated from the first day of treatment to the date of the event or the last follow-up visit. All patients were followed up after the completion of radiotherapy: 1 month after the completion of IMRT, every 3 months in the first 2 years, every 6 months from Year 3 to Year 5, and annually thereafter.

The Statistical Package for Social Sciences, version 17.0 (SPSS Inc., Chicago, IL, USA), software was used for statistical analysis. The χ2, and Fisher exact t tests were used to compare the differences between the extensive skull-base invasion (ESBI) group and limited skull-base invasion (LSBI) group. The local failure free survival (LFFS), distant metastasis free survival (DMFS), progression free survival (PFS), and overall survival (OS) were estimated by use of the Kaplan–Meier method. LFFS, DMFS, PFS and OS were measured from Day 1 of treatment to the date of the event. Multivariate analysis was performed by using the Cox proportional hazards model. All statistical tests were two sided, and *P* < 0.05 was considered to be statistically significant.

## Results

### Grading of MRI-detected skull-base invasion

Incidence of skull-base invasion of each site in the 469 patients is shown in Table 1. Of the 469 patients, 185 patients were classified into the LSBI group, and 284 patients were classified into the ESBI group. The patient characteristics of the LSBI group and ESBI group are shown in Table 2.

**Table 1:** Incidence of Invasion of Each Site in 469 Patients with Skull-base Invasions according to MRI

Site of skull-base invasion	Bilateral	Left	Right	Total (%)
Base of sphenoid	439 (93.6)			439 (93.6)
Foramen lacerum	116 (24.7)	117 (24.9)	95 (20.3)	328 (69.9)
Clivus	239 (51.0)			239 (51.0)
Petrous apex	58 (12.4)	93 (19.8)	85 (18.1)	236 (50.3)
Medial pterygoid plate	11 (2.3)	107 (22.8)	84 (17.9)	202 (43.1)
Foramen ovale	20 (4.3)	92 (19.6)	90 (19.2)	202 (43.1)
Pterygopalatin fossa	12 (2.6)	69 (14.7)	57 (12.2)	138 (29.4)
Foramen rotundum	14 (3.0)	47 (10.0)	52 (11.1)	113 (24.1)
Foramen magnum	93 (19.8)			93 (19.8)
Hypoglossal canal	14 (3.0)	34 (7.2)	41 (8.7)	89 (29.0)
Lateral pterygoid plate	2 (0.4)	36 (7.7)	37 (7.9)	75 (16.0)
Jugular foramen	2 (0.4)	22 (4.7)	29 (6.2)	53 (11.3)

**Table 2:** Patient Characteristics

Characteristic	LSBI group (*N* = 185)	ESBI group (*N* = 284)	P
Sex			
Male	113 (61.1)	209 (73.6)	0.004
Female	72 (38.9)	75 (26.4)	
Age (year)			
< 48	99 (53.5)	127 (44.7)	0.062
≥ 48	86 (46.5)	157 (55.3)	
Pathology classification			
Keratinizing	2 (1.1)	2 (0.7)	0.664
Non-keratinizing	183 (98.9)	282 (99.3)	
T classification			
T3	174 (94.1)	83 (29.2)	< 0.001
T4	11 (5.9)	201 (70.8)	
N classification			
N0	26 (14.1)	29 (10.2)	0.329
N1	74 (40.0)	136 (47.9)	
N2	61 (33.0)	87 (30.6)	
N3	24 (13.0)	32 (11.3)	
Overall stage			
III	152 (82.2)	73 (25.7)	< 0.001
IVA	33 (17.8)	211 (74.3)	
Concurrent chemotherapy			
Yes	176 (95.1)	270 (95.1)	0.975
No	9 (4.9)	14 (4.9)	

*ESBI* extensive skull-base invasion, *LSBI* limited skull-base invasion

### Treatment outcomes

**Fig. 1:**
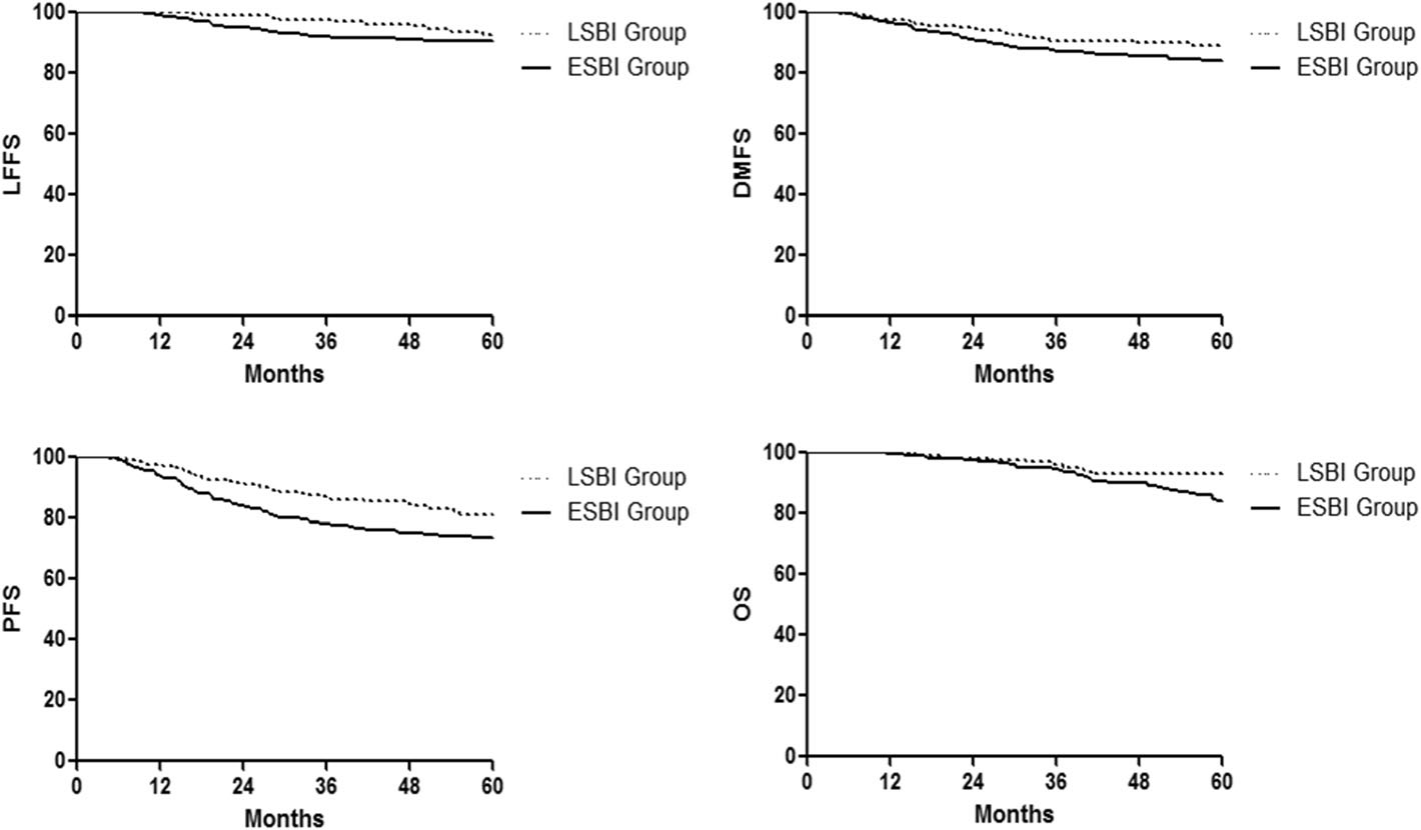
Kaplan–Meier survival curves for patients in the ESBI and LSBI groups. (ESBI, extensive skull-base invasion; LSBI, limited skull-base invasion; LFFS, local failure free survival; DMFS, distant metastasis free survival; PFS, progression free survival; OS, overall survival)

The median follow-up period was 61 months (range, 2–116 months). By the last follow-up, 22.8% (107/469) of patients developed treatment failure and more patients developed treatment failure in the ESBI group (26.1% vs. 17.8%, *p* = 0.038). The details of treatment failure are listed in Table 3. The estimated 5-year LFFS, DMFS, PFS, and OS rates for the whole group were 91.9, 86.1, 76.6 and 87.5%, respectively. The estimated 5-year LFFS, DMFS, PFS, and OS rates for patients in the LSBI and ESBI group were 92.6% versus 90.8% (*P* = 0.296), 90.0% versus 84.2% (*P* = 0.116), 81.3% versus 73.6% (*P* = 0.032), and 93.1% versus 84.0% (*P* = 0.024), respectively (Fig. 1).

**Table 3:** Patterns of Treatment Failure for Patients with Skull-base Invasion after IMRT

Treatment Failure Pattern	LSBI group (*N* = 185)	ESBI group (*N* = 284)	P
Distant only	12 (6.5)	34 (12.0)	0.051
Bone	3 (1.6)	9 (3.2)	
Liver	3 (1.6)	9 (3.2)	
Lung	5 (2.7)	11 (3.9)	
Bone and liver	0	1 (0.4)	
Bone and lung	1 (0.5)	2 (0.7)	
Lung and liver	0	1 (0.4)	
Other	0	1^a^(0.4)	
Regional and distant	4 (2.2)	4 (1.4)	
Local and distant	2 (1.1)	3 (1.1)	
Local, regional, and distant	0	1 (0.4)	
Local only	6 (3.2)	18 (6.3)	
Regional only	6 (3.2)	12 (4.2)	
Local and regional	3 (1.6)	2 (0.7)	
Total	33 (17.8)	74 (26.1)	0.038

*ESBI* extensive skull-base invasion, *LSBI* limited skull-base invasion

^a^ Lung, liver, mediastinal and retroperitoneal lymph nodes, and left adrenal gland

### Univariate and multivariate analyses

The value of various potential prognostic factors including age, sex, skull-base invasion, T classification, N classification and concurrent chemotherapy on predicting LFFS, DMFS, PFS, and OS were evaluated. Univariate analysis by log-rank test showed that skull-base invasion (LSBI vs. ESBI) was associated with PFS (*P* = 0.042), and OS (*P* = 0.024) (Table 4). Multivariate analysis by Cox proportional-hazards model showed that the skull-base invasion (LSBI vs. ESBI) was an independent prognostic predictor of PFS (HR 1.523, 95%CI 1.006–2.306, *P* = 0.047). (Table 5).

**Table 4:** Univariate Analysis of Variables Correlated with Various Clinical Endpoints

Characteristic	5y-LFFS	P	5y-DMFS	P	5y-PFS	P	5y-OS	P
Sex								
Male	89.7	0.052	84.9	0.359	73.7	0.017	86.2	0.301
Female	95.2		88.6		82.7		90.1	
Age (y)								
≥ 48	89.6	0.245	85.9	0.866	75.0	0.646	83.3	0.037
<48	93.2		86.3		78.0		91.5	
Skull-base invasion								
LSBI	92.6	0.296	90.0	0.116	81.3	0.032	93.1	0.024
ESBI	90.8		84.2		73.6		84.0	
T classification								
T3	93.1	0.093	88.5	0.036	79.9	0.042	90.9	0.014
T4	89.5		83.2		72.6		83.2	
N classification								
N0	92.2	0.909	94.1	< 0.001	84.7	0.066	87.7	0.879
N1	90.7		89.3		79.2		89.9	
N2	91.5		84.3		73.6		86.3	
N3	93.7		69.9		66.4		80.8	
Overall Stage (the 8th AJCC)								
III	92.7	0.220	91.1	0.001	81.8	0.008	92.4	0.003
IVa	90.4		81.5		71.9		82.7	
Concurrent chemotherapy								
Yes	91.3	0.585	86.4	0.471	75.8	0.924	87.7	0.706
No	94.1		80.9		76.7		84.7	

*ESBI* extensive skull-base invasion, *LSBI* limited skull-base invasion, *LFFS* local failure free survival, *DMFS* distant metastasis free survival, *PFS* progression free survival, *OS* overall survival

**Table 5:** Multivariate Analysis of Variables Correlated with Various Clinical Endpoints

Endpoint	Item	HR	95% CI	P
DMFS	T3 vs. T4	1.741	1.039–2.916	0.035
	N0–1 vs. N2–3	2.272	1.347–3.829	0.002
PFS	N0–1 vs. N2–3	1.621	1.107–2.374	0.013
	Male vs. Female	0.620	0.392–0.980	0.041
	LSBI vs. ESBI	1.523	1.006–2.306	0.047
OS	T3 vs. T4	1.910	1.079–3.382	0.026

*HR* hazard ratio, *CI* confidence interval, *ESBI* extensive skull-base invasion, *LSBI* limited skull-base invasion, *DMFS* distant metastasis free survival, *PFS* progression free survival, *OS* overall survival

### T-classification category of the grading in patients with T3 classification

**Fig. 2:**
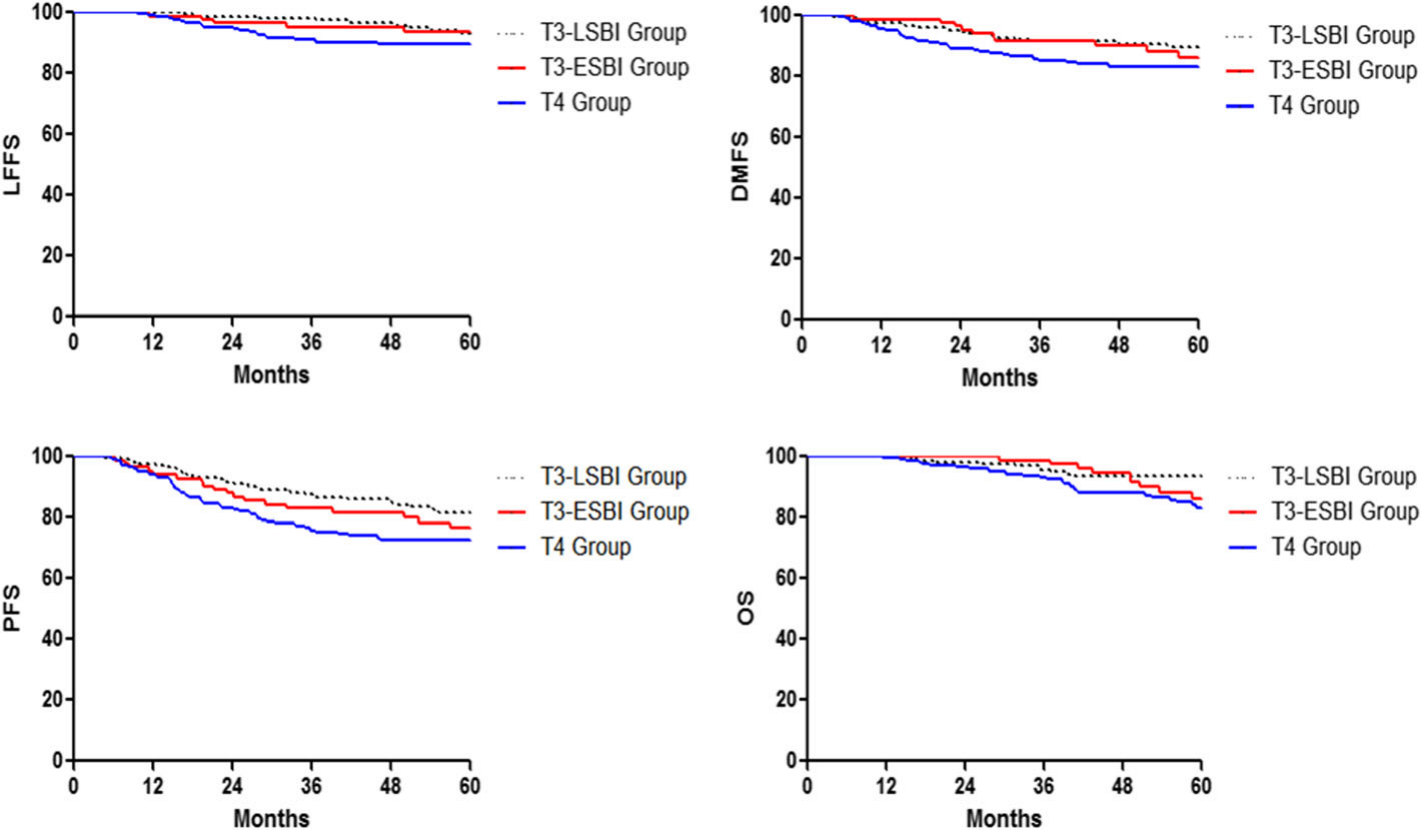
Kaplan–Meier survival curves for patients in the T3-ESBI, T3-LSBI and T4 groups. (ESBI, extensive skull-base invasion; LSBI, limited skull-base invasion; LFFS, local failure free survival; DMFS, distant metastasis free survival; PFS, progression free survival; OS, overall survival)

According to the 8th AJCC staging system, 257 patients were classified as T3 classification. Of these, 83 (32.3%) patients developed extensive skull-base invasion (T3-ESBI) and 174 (67.7%) didn’t (T3-LSBI). The estimated 5-year LFFS, DMFS, PFS, and OS rates for patients in the T3-LSBI and T3-ESBI group were 92.9% versus 93.5% (*P* = 0.997), 89.8% versus 86.1% (*P* = 0.562), 81.6% versus 76.4% (*P* = 0.280), and 93.5% versus 86.3% (*P* = 0.299), respectively. The estimated 5-year LFFS, DMFS, PFS, and OS rates for the patients with T4 classification were 89.5, 83.2, 72.6 and 83.2%, respectively (Fig. 2) No significant difference was observed in terms of LFFS, DMFS, PFS, and OS between patients with T3-ESBI and those with T4 classification (*P* > 0.05). When extensive skull-base invasion was classified as T3 classification, the segregation of survival curves between the T3 and T4 classifications was clearly displayed.

## Discussion

In this study, we observed a high incidence of skull-base invasion in NPC and that grading of skull-base invasion is an independent prognostic factor of PFS in NPC after IMRT.

MRI is recommended as the preferred modalities for NPC staging and has proven to be more sensitive in detecting early infiltration of tumor cells into the bone marrow [8–10]. Based on MRI, skull base erosion may be observed in 50–70% of NPC [4–6, 9, 10]. In this study, 469/695 (67.5%) patients with skull-base invasions were reported.

In the era of IMRT, MRI-detected skull-base invasion was not observed to be an independent prognostic factor for NPC. However, the classification of skull-base invasion (LSBI vs. ESBI) was an independent prognostic factor in T3 (according to the 7th edition of the AJCC staging system) NPC patients in terms of the 5-year OS (*P* = 0.028), DMFS (*P* = 0.032), and PFS rates (*P* = 0.002) [6]. The result of this study indicates that LSBI was associated with a better prognosis in terms of PFS compared to ESBI. Foramen ovale, foramen rotundum, hypoglossal canal and jugular foramen all belong to the ESBI group and are neural foramina. These areas were frequently related with MRI-detected cranial nerve involvement, which was associated with distant metastasis and poor survival [11]. As distant metastasis is the most commonly failure pattern for NPC treated by IMRT, especially, for patients with ESBI [6, 12]. Although most ESBI patients (277/284; 97.5%) in our study were treated by chemoradiotherapy, they still had an unsatisfactory survival rate. Further studies including more intensive systemic approach or newer agents are needed to improve treatment outcome for these patients.

In the 7th edition of the AJCC staging system for NPC, patients with skull-base invasion were classified as T3, and this classification remains in the 8th edition of the AJCC staging system. No significant difference was observed in terms of LFFS, DMFS, PFS, and OS between patients with T3-ESBI and those with T3-LSBI (*p* > 0.05), which was probably associated with the aid of IMRT, MRI, and the use of chemotherapy [12–14]. In addition, when ESBI was classified as T3 classification, the segregation of survival curves between the T3 and T4 classifications was clearly displayed. In a sense, this study demonstrated that it was more suitable for skull-baseinvasion as a single entity to be classified as T3 classification.

There are several limitations in the current study, including the inclusion of patients treated at a single center and the retrospective nature of the study design. The effect of skull-base invasion on the prognosis and staging of patients with NPC should be further confirmed by other cohorts from different centers.

## Conclusion

Grading of MRI-detected skull base erosion is an independent prognostic factor of NPC treated by IMRT. Our results confirm that it is scientific and reasonable for skull-base invasion as a single entity to be classified as T3 classification in the AJCC staging system for NPC.

## Abbreviations

AJCC: American Joint Committee on Cancer
CI: Confidence interval
CT: Computed tomography
DMFS: Distant metastasis free survival
ESBI: Extensive skull-base invasion
HR: Hazard ratio
IMRT: Intensity-modulated radiotherapy
LFFS: Local failure free survival
LSBI: Limited skull-base invasion
NPC: Nasopharyngeal carcinoma
OS: Overall survival
PFS: Progression free survival
PTV: Planning target volume
